# Serum 25-hydroxyvitamin D concentrations in dogs with gallbladder mucocele

**DOI:** 10.1371/journal.pone.0244102

**Published:** 2020-12-16

**Authors:** Jared A. Jaffey, Jodi Matheson, Kate Shumway, Christina Pacholec, Tarini Ullal, Lindsay Van den Bossche, Hille Fieten, Randy Ringold, Keun Jung Lee, Amy E. DeClue

**Affiliations:** 1 Department of Specialty Medicine, Midwestern University College of Veterinary Medicine, Glendale, Arizona, United States of America; 2 Department of Veterinary Medicine and Surgery, Veterinary Health Center, University of Missouri, Columbia, Missouri, United States of America; 3 Department of Clinical Sciences, College of Veterinary Medicine and Biomedical Sciences, Colorado State University, Fort Collins, Colorado, United States of America; 4 Department of Clinical Sciences of Companion Animals, Faculty of Veterinary Medicine, Utrecht University, Yalelaan, Utrecht, The Netherlands; 5 VDI Laboratory, LLC, Simi Valley, California, United States of America; 6 Department of Pathology and Population Medicine, Midwestern University College of Veterinary Medicine, Glendale, Arizona, United States of America; University of Lincoln, UNITED KINGDOM

## Abstract

Gallbladder mucocele (GBM) is a common biliary disorder in dogs. Gallbladder hypokinesia has been proposed to contribute to its formation and progression. The specific cause of gallbladder stasis in dogs with GBM as well as viable treatment options to resolve dysmotility remains unknown. Vitamin D deficiency is one of the many potential causes of gallbladder hypokinesia in humans and repletion results in complete resolution of stasis. Improving our understanding of the relationship between serum vitamin D and GBM could help identify dogs as a model for humans with gallbladder hypokinesia. Furthermore, this relationship could provide insight into the pathogenesis of GBM and support the need for future studies to investigate vitamin D as a novel treatment target. Therefore, goals of this study were i) to determine if serum 25-hydroxyvitamin(OH)D concentrations were decreased in dogs with GBM, ii) if serum 25(OH)D concentrations were different in clinical versus dogs subclinical for GBM, and iii) to determine if serum 25(OH)D concentrations could predict the ultrasonographic type of GBM. Sixty-two dogs (clinical, n = 26; subclinical, n = 36) with GBM and 20 healthy control dogs were included in this prospective observational study. Serum 25(OH)D concentrations were measured with a competitive chemiluminescence immunoassay. Overall, dogs with GBM had lower serum 25(OH)D concentrations than control dogs (P = 0.004). Subsequent subgroup analysis indicated that this difference was only significant in the subclinical group compared to the control dogs (P = 0.008), and serum 25(OH)D concentrations did not significantly differ between dogs clinical for GBM versus subclinical or control dogs, indicating that inflammatory state in clinical dogs was not the major constituent of the observed findings. Decreasing serum 25(OH)D concentrations, but not clinical status, was associated with a more advanced developmental stage of GBM type determined by ultrasonography. Our results indicate that vitamin D has a role in dogs with GBM. Additional studies are needed to assess if reduced vitamin D in dogs with GBM is a cause or effect of their biliary disease and to investigate if vitamin D supplementation could be beneficial for dogs with GBM.

## Introduction

Gallbladder mucocele (GBM) is one of the most common biliary disorders in dogs. This disease is characterized by aberrant secretion of thick mucus by the gallbladder epithelium that can result in life-threatening extrahepatic bile duct obstruction, cholecystitis, and biliary rupture [1]. Gallbladder hypokinesia has been proposed to have a contributory role in the formation and progression of GBM [2]. Gallbladder hypokinesia is defined as gallbladder hypomotility in response to normal physiologic stimuli and results in pathogenic alterations to bile including supersaturation of cholesterol and hydrophobic bile acids [3, 4]. Exposure of gallbladder epithelial and smooth muscle cells to concentrated biliary constituents causes hypersecretion of mucus and inflammation via oxidative damage (H_2_O_2_) and lipid peroxidation; the consequences of which results in the development of gallbladder sludge, mucus plugs, gallstones, cholecystitis, and biliary colic [5–10]. Biliary stasis has been linked to many common conditions in humans including obesity, hyperglycemia, hypothyroidism, hypertriglyceridemia, acalculous cholecystitis, pregnancy, and vitamin D deficiency [11–16].

While impaired gallbladder motility is postulated to have a role in GBM formation, the specific disease etiology remains unknown. Affected dogs have a high incidence of concurrent endocrinopathies (i.e., hyperadrenocorticism, hypothyroidism, and diabetes mellitus) as well as hypertriglyceridemia and hypercholesterolemia [17–19]. Further, purebred dogs including Shetland Sheepdogs, Miniature Schnauzers, Border Terriers, Cocker Spaniels, Beagles, Chihuahuas, and Pomeranians are overrepresented [19–21]. These associations with breed as well as primary hyperlipidemia or endocrine related secondary hyperlipidemia indicate that both genetic and metabolic factors influence disease pathogenesis.

Antemortem diagnosis of GBM formation is easily achieved with abdominal ultrasonogram [22]. The ultrasonographic classification of GBM in dogs has evolved since it was first described 20 years ago [23]. Recently, Choi et al. (2014) described 6 different types of GBM based on unique ultrasonographic patterns. This classification scheme combined with widespread use of ultrasonography allows for earlier recognition and intervention in dogs with GBM. The Choi et al. (2014) study found an association between GBM type II and gallbladder rupture, which a subsequent study demonstrated that GBM rupture was associated with significantly greater risk of death [20]. Further, a recent retrospective study revealed that GBM type was associated with survival in a multivariable model [24].

Dogs with GBM that demonstrate clinical signs attributable to biliary tract disease require emergent surgical removal of the gallbladder. The perioperative mortality rate in these dogs range from 7–45% [19, 20, 24–29]. In subclinical dogs, there is a paucity of published information available for effective medical options to resolve or even mitigate progression of GBM. Ursodeoxycholic acid is commonly used in dogs subclinical for GBM because of its ability to induce choleresis and lower the concentration of hydrophobic bile acids. However, there is a dearth of published data that demonstrate a clear clinical benefit in these dogs. In contrast, various prokinetic drugs in humans such as cisapride, erythromycin, cholestyramine, prostigmine, and bethanachol have been shown to improve gallbladder ejection fraction and decrease accumulation of gallbladder sludge [13]. Further, a recent abstract in humans with gallstones found that parenteral vitamin D administration resolved gallbladder stasis [30]. The ligation of vitamin D with its cytosolic receptor results in increased protein synthesis as well as phosphorylation of intracellular proteins enhancing the kinetics of smooth muscle contraction [31–33]. In addition, activated vitamin D receptors in mice and humans increase hepatic expression of CYP3A4, a cytochrome P450 enzyme that decreases bile acid hydrophobicity, and thus toxicity, of bile acids [34–37].

Dogs could be an ideal animal model for studies that investigate the effect of vitamin D supplementation on gallbladder motility. The human literature is limited to a single study that investigated parenteral vitamin D supplementation in pregnant women [30]. Dogs have similar co-morbidities (e.g., endocrinopathies, hyperlipidemia, cholecystitis), and surgical interventions for biliary disease. Likewise, vitamin D in dogs, like humans, has potent immunologic effects and can predict outcome in various diseases highlighting an overlap in vitamin D physiology [38–42]. There are several differences in vitamin D metabolism that would strengthen the use of dogs as a model. Dogs are not able to synthesize vitamin D in their skin in response to ultra-violet light from the sun, and thus are entirely reliant on diet to fulfill their vitamin D needs [43].

Improving our understanding of the relationship between serum vitamin D and GBM could help identify dogs as a viable model for humans with gallbladder hypokinesia. Vitamin D concentrations in dogs are not influenced by age, sex, body condition, or season; therefore, studies focused on vitamin D supplementation will not be confounded by variables inherent to humans [44–49]. Furthermore, this relationship could provide insight into the pathogenesis of GBM and support the need for future studies to investigate vitamin D as a novel treatment target. Therefore, this study had three main objectives: i) to determine if dogs with GBM have decreased serum 25-hydroxyvitamin (OH)D, the main circulating analogue of vitamin D, ii) assess if serum 25(OH)D concentrations were different in clinical versus dogs subclinical for GBM, and iii) to determine if serum 25(OH)D concentration could predict the ultrasonographic type of GBM in dogs. This information is important because it could support that lower vitamin D concentrations exacerbate gallblader hypomotility and thus lead to a more advanced developmental stage of GBM. We hypothesized that serum 25(OH)D concentration would be lower in dogs with GBM compared to healthy control dogs and that differences would be found in multi-group comparisons (i.e., clinical for GBM, subclinical for GBM, and controls). We also hypothesized that serum 25(OH)D concentrations would be predictive of ultrasonographic GBM type.

## Materials and methods

### Criteria for selection of cases and study design

Dogs that were diagnosed with a GBM via ultrasonography, gross appearance/ histopathology, or both, at the Midwestern University College of Veterinary Medicine (MWU-CVM), University of Missouri Veterinary Health Center (MU-VHC), Colorado State University College of Veterinary Medicine (CSU-CVM), and Utrecht University Faculty of Veterinary Medicine (UU-FVM) between July 2018 and November 2019 were eligible for inclusion in this prospective observational study. Dogs were included in the study after obtaining informed owner consent. This study was conducted in accordance with guidelines for clinical studies and approved by each institutions’ Animal Care and Use Committee (MWU-CVM, #2925; MU-VHC, #7334; CSU-CVM, VCS#2019–203). Approval from the Animal Care and Use Committee from UU-FVM was not required because only dogs with serum left-over from other diagnostic testing were included. Exclusion criteria were pregnancy, lactation, history of chronic kidney disease, supplementation with vitamin D or calcium, or diagnosis of hypercalcemia of malignancy, hyperparathyroidism, or hypoparathyroidism. Pre-analytical factors that warranted exclusion included moderate to marked serum hemolysis. Dogs were also excluded if a concurrent, clinically relevant comorbid disease was diagnosed. Exclusionary diseases included those with a known (e.g., chronic enteropathy, diabetic ketoacidosis, neoplasia, congestive heart failure, immune-mediated diseases) or suspected (e.g. pancreatitis, cirrhosis) effect on serum 25(OH)D concentrations and were determined on a case-by-case basis by a board-certified small animal internist (JAJ). A second population of healthy control dogs were included after review of clinical history, physical examination, complete blood count, serum biochemical profile, and urinalysis by a board-certified small animal internist (JAJ). Control dogs could not have had any illnesses, vaccination, or been administered any medications, except monthly parasiticides within 60 days of enrollment. For each control dog, ultrasonography was used to confirm the absence of GBM formation based on anechoic contents or small volume of gravity dependent sludge.

Gallbladder mucoceles were identified in one of two ways; ultrasonography or gross and histologic evaluation of the gallbladder. To be diagnosed via ultrasonography, images had to be captured by a board-certified veterinary radiologist, a veterinary radiology resident under the direct supervision of a board-certified veterinary radiologist, a registered diagnostic medical/veterinary sonographer, or a board-certified small animal internist trained in ultrasonography. Gallbladder mucocele was diagnosed with ultrasonography based on a gallbladder containing gravity independent, immobile material [20]. Then the static images and video recordings (when available) were reviewed by two board-certified veterinary radiologists (JM, KS) and assigned a consensus GBM type that ranged from type I-to-type VI based on previously established criteria [22]. Serum 25(OH)D concentrations were not known by investigators at the time abdominal ultrasonograms were performed. A GBM diagnosis could also be made if the gross and histologic evaluation post-cholecystectomy or post-mortem examination was consistent with a distended gallbladder with an abnormal accumulation of inspissated, amorphous mucus in combination with histologic evidence of cystic mucosal hyperplasia/hypertrophy (S1 Fig) [20]. A diagnosis of gallbladder rupture was made based on previously reported ultrasonographic and intraoperative criteria [20]. Dogs were considered to be clinical or subclinical for GBM based on the presence or absence, respectively of ≥ 1 of the following clinical signs reported by the dog owner to have occurred within the 7 days preceding presentation: vomiting, lethargy, anorexia, hyporexia, abdominal pain or distension, jaundice, diarrhea, hypodipsia. Board-certified small animal internists from each respective institution determined if clinical signs were directly related to GBM.

### Sample collection and serum 25(OH)D measurement

Medical records were reviewed for each dog enrolled. The age, sex, and breed were recorded for each. The presence of clinical signs was identified for each dog at the time of hospital admission when available. Other data retrieved from medical records included total white blood cell count, total blood bilirubin concentration, description of clinical signs, pursuit of cholecystectomy, presence of gallbladder rupture, survival to hospital discharge, and cause of death or euthanasia. Blood samples for serum 25(OH)D measurement were collected into serum separator tubes, centrifuged, and serum removed within 1 hour of collection. These samples were obtained preoperatively in those dogs that subsequently had a cholecystectomy. The serum was placed in freezer-resistant plastic tubes and stored at -80°C for < 24 months for batch analysis. Serum vitamin D stored frozen at -80°C remains stable in humans for many years and is believed to be consistent across species [50]. Samples were packed with dry ice and shipped overnight to VDI laboratory for serum 25(OH)D quantification. Tubes were coded so that the identity of the sample was known only by the investigators and not the laboratory measuring 25(OH)D. Serum 25(OH)D concentrations were determined using a competitive chemiluminesence immunoassay as previously described [51]. This assay has an intra- and inter-assay precision (five replicates) of 4.0 and 3.4%, respectively. Freeze–thaw testing for 25(OH)D stability revealed there was no statistically significant difference for up to four freeze–thaw cycles.

### Statistical analysis

Statistical analysis was performed with commercial software (SigmaPlot, Systat Software). A Shapiro-Wilk test was used to assess normality. Categorical data were presented as number, proportions, or percentages, as appropriate. Non-normally distributed data were described as median and (interquartile range [IQR]: 25^th^ quartile to 75^th^ quartile). Analyses of total white blood cell count and total blood bilirubin concentration were made based on the fold change with respect to the upper limit of the corresponding reference interval. Data are graphically illustrated using box and whisker plots with outliers defined using the Tukey method in which outliers are values 1.5×IQR below or above the 25^th^ and 75^th^ quartiles, respectively. Outliers were not excluded from statistical analyses. Serum 25(OH)D concentrations were compared between dogs with GBM and controls using a Mann-Whitney rank sum test. A Kruskal-Wallis One Way Analysis of Variance on Ranks test was used to compare serum 25(OH)D concentrations among dogs clinical for GBM, dogs subclinical for GBM, and controls. Dunn’s test of multiple comparisons using rank sums was used to evaluate multiple pairwise comparisons for significant Kruskal-Wallis tests. Univariate logistic regression analyses were performed to investigate the association between ultrasonographic GBM type and serum 25(OH)D concentration (continuous variable). The binary dependent variables in these analyses were type I GBM (yes/no), type II GBM (yes/no), type III GBM (yes/no), type IV GBM (yes/no), type V GBM (yes/no), and type IV and V GBM (yes/no). To explore the association between GBM type and clinical status (clinical/subclinical), univariate analysis by means of the chi-square or fisher-exact test were performed. The level of significance was set at P < 0.05.

## Results

### Animal population

A total of 95 dogs from four academic veterinary hospitals were eligible for inclusion in this study. Twelve dogs were excluded because of comorbid diseases including neoplasia staging (n = 4), intermittent gastrointestinal signs (n = 4), one each of severe pancreatitis, diabetic ketoacidosis, cirrhosis, and a lytic bone lesion. One dog was excluded because marked serum hemolysis precluded 25(OH)D quantification, leaving 82 dogs, including control dogs (n = 20), that were ultimately included in the study.

A total of 62 purebred dogs and 20 mixed breed dogs were included. Purebred dogs included Chihuahua (n = 8), Labrador Retriever (4), Maltese (4), Beagle (4), Shetland Sheepdog (3), German Shepherd Dog (3), Cocker Spaniel (2), Springer Spaniel (2), Toy Poodle (2), Yorkshire Terrier (2), Miniature Poodle (2), Miniature Schnauzer (2), Cavalier King Charles Spaniel (2), Chow Chow (2), Pit Bull Terrier (2), Rat Terrier (2), and one each of Bichon Frise, Australian Cattle Dog, Catahoula Leopard Dog, Dachshund, English Cocker Spaniel, Great Dane, Jack Russell Terrier, Parson Russell Terrier, Pomeranian, Miniature Pinscher, Miniature Dachshund, Pug, Queensland Heeler, West Highland White Terrier, Scottish Deerhound, and Shih Tzu. The median age was 11 years (IQR, 8.2–13.2). The study included 42 spayed females, 34 castrated males, and 3 each of intact females and males.

**Fig 1:**
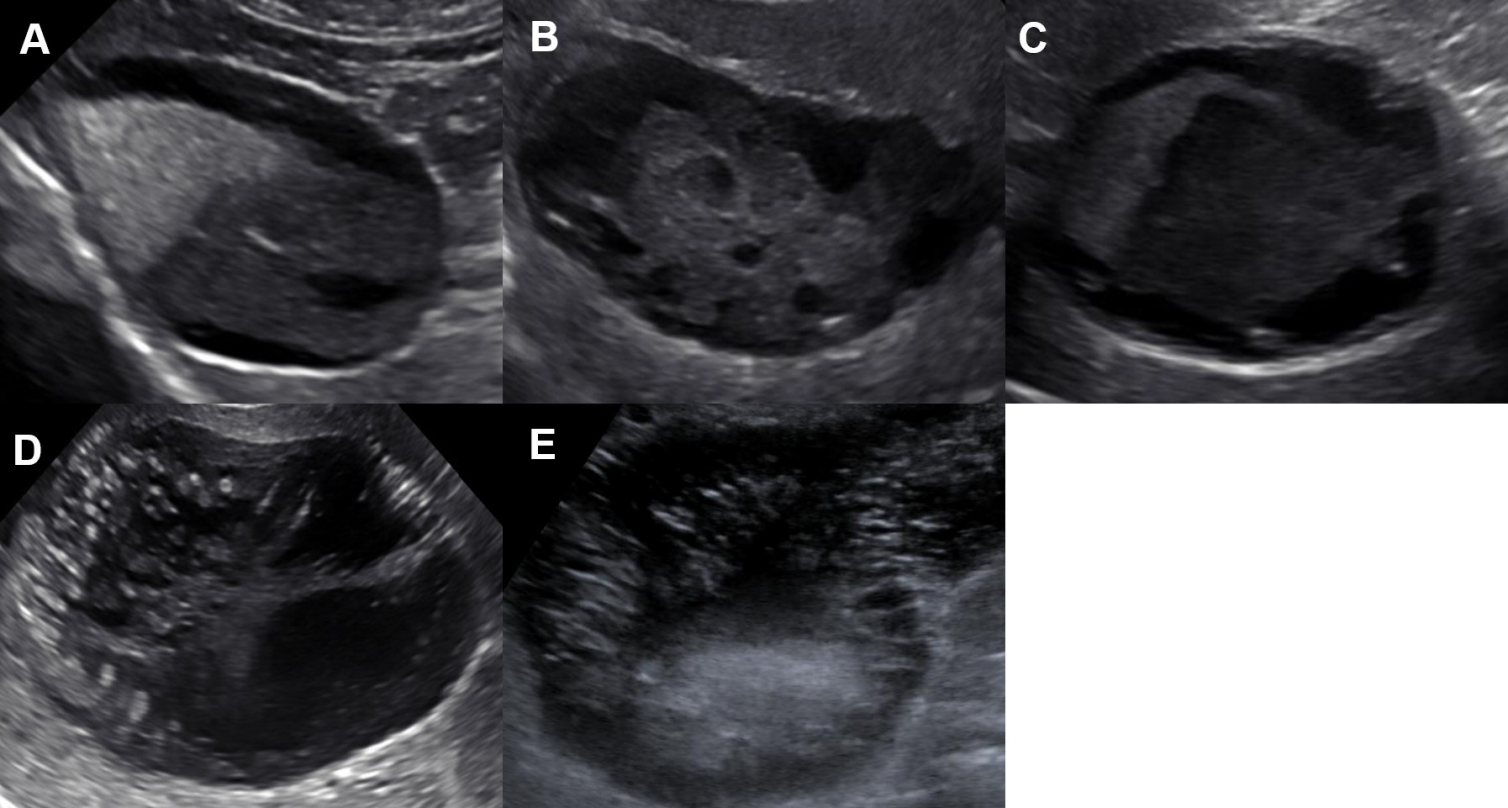
Representative ultrasonographic static images of gallbladder mucocele types obtained from this study population (adapted from Choi et al., 2014) [22]. (A) type I: organized, gravity independent, echogenic debris filling >30% of gallbladder lumen; (B) type II: combination of organized echogenic debris with partial stellate strands adhered to the gallbladder wall; (C) type III: stellate pattern; (D) type IV: combination of stellate and kiwi pattern; (E) type V: kiwi pattern with echogenic debris.

There were 62 dogs with GBM of which 26 (42%) were clinical and the remaining 36 (58%) dogs were subclinical. Descriptive demographic data from dogs with GBM including subgroups (i.e., clinical and subclinical) as well control dogs can be found in Table 1 and breed distribution in S1 Table. The reason that abdominal ultrasonogram examinations were performed in subclinical dogs included increased liver enzyme concentrations (n = 19), recheck previously identified GBM (8), hyperadrenocorticism differentiation [pituitary dependent versus functional adrenocortical tumor] (5), polyuria/polydipsia (3), and a rectal stricture in one dog. All 62 dogs had an ultrasonographic diagnosis of GBM. A definitive GBM type could not be confirmed in one dog based on the limited number of available static images. The most commonly identified ultrasonographic GBM type was type II (n = 25; 40%), followed by type IV (16; 26%), and type I (12; 19%) (Fig 1; Table 2). All 19 dogs with gross/histopathologic data available confirmed the initial ultrasonographic GBM diagnosis. All but one of those dogs had an assigned ultrasonographic GBM type. Detailed distribution of ultrasonographic GBM type can be found in Table 2.

**Table 1:** Demographic data for dogs with gallbladder mucoceles (clinical and subclinical combined), dogs clinical for gallbladder mucocele, dogs subclinical for, and healthy control dogs without gallbladder mucocele.

Variable	Gallbladder mucocele (total)	Clinical gallbladder mucocele	Subclinical gallbladder mucocele	Controls
Number	62	26	36	20
Age (years)^a^	12.0 (10.4–13.5)	11.7 (10.3–13.3)	12.3 (10.4–13.5)	5.3 (1.4–9.0)
Sex (FS, FI, MN, MI)	33, 0, 27, 2	13, 0, 12, 1	20, 0, 15, 1	9, 3, 7, 1

^a^Data presented as median (interquartile range).

FS, female-spayed; FI, female-intact; MN, male-neutered; MI, male-intact.

**Table 2:** Distribution of ultrasonographic type of gallbladder mucocele in all dogs with gallbladder mucocele, based on clinical status (i.e., clinical and subclinical), and dogs with gross/histopathology. Data displayed as number (%).

Variable	Gallbladder mucocele	Clinical gallbladder mucocele	Subclinical gallbladder mucocele	Gross/histopathology
Number	62	26	36	19
Non-classified type (%)	1 (2)	1 (4)	0 (0)	1 (5)
Type I (%)	12 (19)	6 (23)	6 (17)	5 (26)
Type II (%)	25 (40)	8 (31)	17 (47)	5 (26)
Type III (%)	5 (8)	4 (15)	1 (3)	3 (17)
Type IV (%)	16 (26)	7 (27)	9 (25)	5 (26)
Type V (%)	3 (5)	0 (0)	3 (8)	0 (0)
Type VI (%)	0 (0)	0 (0)	0 (0)	0 (0)

### Clinical features of dogs clinical for gallbladder mucocele

A total of 85% (22/26) of dogs demonstrated ≥ 2 clinical signs. The median number of clinical signs exhibited per dog was 3 signs (IQR, 2.0–5.0). The most commonly owner reported signs included lethargy (n = 19; 73%), vomiting (17; 65%), hyporexia (11; 42%), anorexia (8; 31%), jaundice (7; 27%), abdominal discomfort/pain (6; 23%), and diarrhea (6; 23%). Most dogs (25; 96%) clinical for GBM had total white blood count measured. Leukocytosis was identified in 48% (12/25) of dogs and the median fold change was 1.09 times the upper limit of the reference interval (IQR, 0.70.-1.49). Seventy-three percent (19/26) of dogs clinical for GBM had total blood bilirubin concentration measured. The median total blood bilirubin concentration was 0.9 times the upper limit of the reference interval (IQR, 0.50–26.00). Seventy-three percent (19/26) of dogs had a cholecystectomy. Nineteen percent (5/26) of dogs had gallbladder rupture confirmed via ultrasonography, intraoperatively, or both. A total of 31% (8/26) of dogs did not survive to hospital discharge. Two dogs died and 6 were euthanized. The reason for euthanasia included poor prognosis (2), and one each of the following: sepsis with multiple organ dysfunction syndrome, sepsis with acute kidney injury, septic bile peritonitis, and bile peritonitis.

### Vitamin D, clinical status, and ultrasonographic gallbladder mucocele type

**Fig 2:**
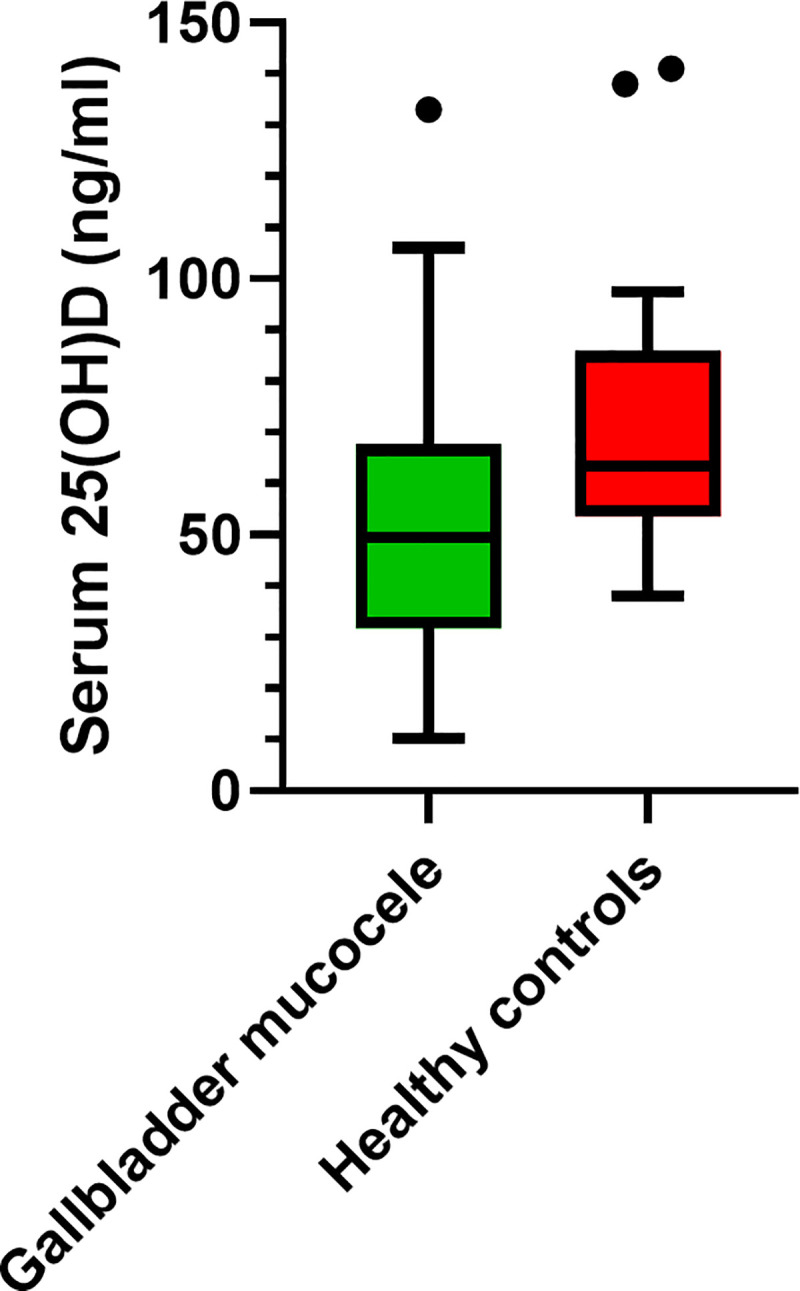
Box and whisker plot comparing serum 25-hydroxyvitamin (OH)D concentrations in dogs with gallbladder mucocele and healthy control dogs without gallbladder mucocele. The top and bottom of the boxes represent the 25^th^ and 75^th^ quartiles, respectively with the black horizontal line representing the median. The whiskers extend up to 1.5×IQR below and above the 25^th^ and 75^th^ quartiles, respectively. Closed circles above the whiskers represent outlier values. Dogs with gallbladder mucocele (n = 62) had lower serum 25(OH)D concentration than healthy control dogs (n = 20; P = 0.004).

**Fig 3:**
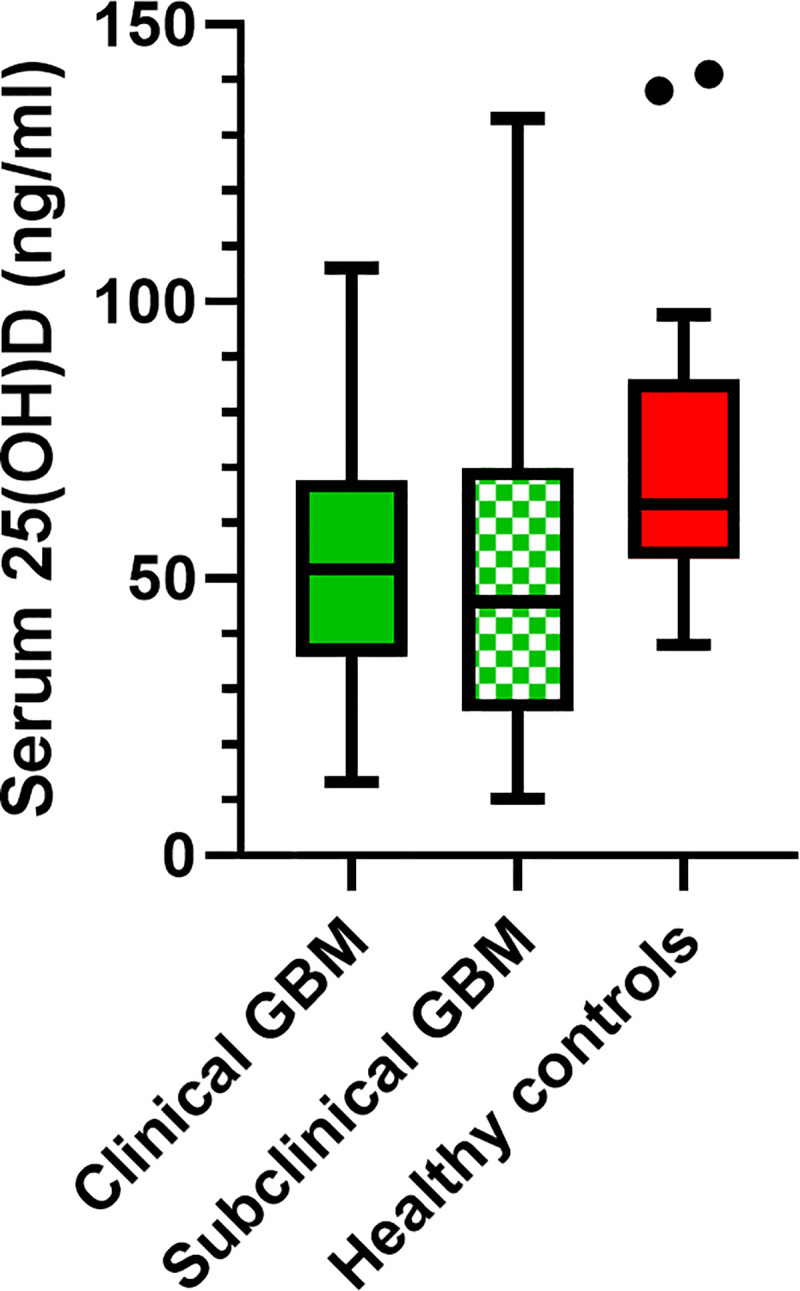
Box and whisker plot comparing serum 25-hydroxyvitamin (OH)D concentrations in dogs clinical for gallbladder mucocele (GBM), subclinical for GBM, and healthy control dogs. The top and bottom of the boxes represent the 25th and 75th quartiles, respectively with the black horizontal line representing the median. The whiskers extend up to 1.5×IQR below and above the 25th and 75th quartiles, respectively. Closed circles above the whiskers represent outlier values. Post-hoc pairwise multiple comparisons revealed that dogs subclinical for GBM had lower serum 25(OH)D concentrations than control dogs (P = 0.008). There was no difference in serum 25(OH)D concentration between dogs clinical for GBM and those that were subclinical (P = 1.00) or healthy control dogs (P = 0.14).

Dogs with GBM had significantly lower serum 25(OH)D concentrations than healthy control dogs (P = 0.004; Fig 2). Next, a subgroup comparison of serum 25(OH)D concentration was performed to determine if clinical status (i.e., clinical or subclinical) contributed to the reason serum 25(OH)D concentrations were lower in dogs with GBM compared to control dogs. There was an overall difference in serum 25(OH)D concentration between the three groups (clinical, subclinical, and control dogs; P = 0.010). Subsequent pairwise multiple comparisons revealed that dogs subclinical for GBM had lower serum 25(OH)D concentrations than control dogs (P = 0.008), but differences between dogs clinical for GBM and dogs subclinical for GBM or control dogs were not identified (Fig 3).

Next, serum 25(OH)D (continuous variable) was evaluated in a univariate logistic regression analysis to determine if it was predictive of the ultrasonographic type of GBM. Logistic regression analyses indicated that serum 25(OH)D concentration was associated with an ultrasonographic diagnosis of a type I as well as type IV GBM. For every 10 ng/ml unit decrease in serum 25(OH)D concentration the odds that a dog with a GBM was diagnosed as type I decreased by 27% and the odds for a type IV increased by 28% (Table 3). There was also an association between serum 25(OH)D concentration and ultrasonographic type of GBM when the dependent variable was dichotomized. These results indicate that for every 10 ng/ml unit decrease in serum 25(OH)D concentration the odds that a dog with a GBM was diagnosed with a type IV or V GBM increased by 25% (Table 3). Clinical status was not associated with ultrasonographic GBM type in any of the chi-square or fisher-exact tests (Table 3).

**Table 3:** Association of serum 25-hydroxyvitamin D and clinical status with ultrasonographic type of gallbladder mucocele.

**Type I gallbladder mucocele**			
**Variable**	**Odds ratio**	**95% CI**	**P-value**
Serum 25(OH)D concentration	1.027	1.002–1.054	0.03
Clinical status (clinical/subclinical)	0.633	0.178–2.253	0.53
**Type II gallbladder mucocele**			
**Variable**	**Odds ratio**	**95% CI**	**P-value**
Serum 25(OH)D concentration	1.007	0.987–1.027	0.49
Clinical status (clinical/subclinical)	0.526	0.181–1.526	0.36
**Type III gallbladder mucocele**			
**Variable**	**Odds ratio**	**95% CI**	**P-value**
Serum 25(OH)D concentration	0.978	0.937–1.021	0.31
Clinical status (clinical/subclinical)	6.667	0.698–63.702	0.15
**Type IV gallbladder mucocele**			
**Variable**	**Odds ratio**	**95% CI**	**P-value**
Serum 25(OH)D concentration	0.972	0.946–0.999	0.04
Clinical status (clinical/subclinical)	1.167	0.368–3.699	0.97
**Type V gallbladder mucocele**			
**Variable**	**Odds ratio**	**95% CI**	**P-value**
Serum 25(OH)D concentration	0.996	0.951–1.044	0.87
Clinical status (clinical/subclinical)	0.000	0.000-infinity	0.26
**Type IV and V gallbladder mucocele**			
**Variable**	**Odds ratio**	**95% CI**	**P-value**
Serum 25(OH)D concentration	0.975	0.950–0.999	0.04
Clinical status (clinical/subclinical)	0.778	0.255–2.371	0.87

CI, confidence interval; 25-hydroxyvitamin (OH)D.

## Discussion

In our investigation, we found that dogs with GBM had significantly lower serum 25(OH)D concentrations than healthy control dogs and a subsequent subgroup analysis indicated that this difference was only significant in subclinical dogs compared to controls. Further, we found that serum 25(OH)D concentration, but not clinical status, was associated with the developmental stage of GBM determined by ultrasonography.

The majority of circulating vitamin D is bound to vitamin D binding protein (70–80%) or albumin (10–20%), both of which are negative acute phase proteins that decrease with systemic inflammatory stimuli and result in renal loss of filtered unbound vitamin D [52, 53]. These physiologic changes result in decreased circulating vitamin D concentrations in dogs and humans with critical illness [38, 54]. With this in mind, we expected a more severe decrease in serum 25(OH)D concentration in dogs clinical for GBM. While dogs with GBM had significantly lower serum 25(OH)D concentrations than healthy control dogs, our subgroup analysis indicated that dogs clinical for GBM did not have lower vitamin D concentrations compared to subclinical or control dogs. The serum 25(OH)D concentrations in control dogs included in this study (median, IQR; 63.4 ng/ml, 53.6–86.0) were similar to those previously reported by VDI laboratory in a study comprised of 282 healthy control dogs (68.9 ng/ml, 54.8–87.9) [51].

While unexpected, the lack of a significant decrease in vitamin D in the clinical GBM group suggests the possibility that decreased vitamin D concentrations in dogs with GBM are not exclusively a consequence of systemic illness. Instead, decreased vitamin D concentrations might have a causal role in the pathophysiology of gallbladder hypokinesia and GBM formation, progression, or both in dogs. Decreased circulating vitamin D could also be the result of GBM because of cholestasis induced fat malabsorption and poor vitamin D uptake from the gut. The plausibility of fat malabsorption being the key driver of lower circulating vitamin D in dogs with GBM is questionable for two reasons. First, in dogs with exocrine pancreatic insufficiency, a fat maldigestive disease with severe malabsorption, only 5% (1/20) of dogs had low serum 25(OH)D concentrations suggesting that fat malabsorption must be severe before vitamin D deficiency develops in dogs [55]. Second, dogs that were subclinical for GBM had significantly lower serum 25(OH)D concentrations than healthy control dogs, yet they had no overt features of fat malabsorption. One would expect clinical signs of fat malabsorption (e.g. diarrhea, steatorrhea, weight loss, or poor body condition) if moderate to severe disease was present. Notably, subclinical dogs by definition, did not demonstrate any of the aforementioned clinical signs. Additional investigation pertaining to whether the reduction in circulating vitamin D contributes to GBM formation/progression or is a sequela of this biliary disease is needed.

Gallbladder hypokinesia has been proposed to have a contributory role in the pathogenesis of GBM in dogs [2]. Impaired gallbladder emptying results in prolonged exposure of the gallbladder epithelium to concentrated toxic hydrophobic bile acids causing inflammation and mucus hypersecretion [56]. Vitamin D deficiency in humans is a risk factor for gallbladder hypokinesia and repletion reconciles gallbladder motility. This further supports that vitamin D deficiency is likely a cause rather than a consequence of gallbladder hypokinesia in humans [13, 30]. The results in the current study suggest the same could be true in dogs, as we found that decreasing serum vitamin D concentrations were associated with greater odds that a dog would have a more advanced developmental stage of GBM. One explanation for this relationship is that lower serum vitamin D concentrations magnified gallbladder stasis resulting in a more advanced (i.e. higher) GBM type. However, we cannot entirely exclude the possibility that higher GBM types yielded more severe cholestasis and with it, enhanced malabsorption of fat-soluble vitamins (A, D, E, and K). Future studies in which the effect of vitamin D administration has on gallbladder ejection fraction in dogs with and without GBM are needed to further investigate our hypothesis that vitamin D influences gallbladder motility.

A major limitation of this study was that it was a prospective observational study; therefore, definitive cause and effect conclusions could not be determined. Dogs in this study were screened with physical examinations, routine clinicopathologic diagnostic tests, and ultrasonogram examinations for clinically relevant disorders that could have affected circulating vitamin D concentrations and were excluded if identified. However, it is possible that some dogs with GBM had occult disease processes; such as neoplasia, inflammatory, or infectious diseases that could have lowered serum 25(OH)D concentrations. Further, clinical signs associated with GBM overlap with many diseases; therefore, it is possible that in some cases clinical signs were not definitively caused by GBM. Clinical and clinicopathologic data were included to provide context as to the severity of illness and systemic inflammation in dogs clinical for GBM. However, objective markers of inflammation (e.g., c-reactive protein, fibrinogen, haptoglobin) and/or illness severity scores (e.g., acute patient physiologic ad laboratory evaluation) would have more accurately captured this. Future studies investigating the role of vitamin D in dogs with GBM should include these aforementioned markers of inflammation and illness severity scoring system. Ultrasonogram video recordings allow for more accurate GBM type classifications than static images because they provide a global assessment of the gallbladder while static images capture only portions. Therefore, the use of static images alone could have led to the misidentification of GBM type in some dogs. The lack of association between GBM type and clinical status might not have been adequately powered to identify an association if one were present (type II error). However, our results are corroborated by a previous study that determined GBM type was not associated with clinical status [22]. Serum was kept frozen at -80°C for a maximum of 24 months before quantification of 25(OH)D concentrations. The long-term stability of serum 25(OH)D in dogs has not been published but is believed to similar to humans in which 25(OH)D concentrations have been shown to remain stable frozen in serum and plasma for many years [50]. Information about the diet that each dog was offered was not routinely recorded. Vitamin D was only measured at a single time point and the association between dietary intake of vitamin D, circulating vitamin D concentrations and GBM were not assessed. Future investigations should consider evaluating these associations.

## Conclusions

Our findings reveal that dogs with GBM have significantly lower serum 25(OH)D concentrations than healthy control dogs and the lack of difference between dogs clinical for GBM versus subclinical or control dogs, indicate that the inflammatory state in clinical dogs was not the major constituent of the observed finding. In addition, decreasing serum 25(OH)D concentrations, but not clinical status, was associated with a more advanced developmental stage of GBM type determined by ultrasonography. Additional studies are needed to assess if reduced vitamin D in GBM dogs is a cause or effect of biliary disease and investigate if vitamin D supplementation could be beneficial for dogs with GBM.

## Supporting information

S1 Table Breed distribution in dogs with gallbladder mucocele and healthy control dogs. (DOCX)

S1 Fig **Gross (A) and histopathologic (B) image of a gallbladder mucocele in a dog.** (A) The gallbladder is markedly expanded with a large amount of tenacious to gelatinous, mucinous, yellow to dark purple bile; (B) The mucosal epithelial cells are proliferative forming glandular and frond like structures that are lined by cuboidal to tall columnar epithelial cells. The mucosa and the luminal surface are filled with eosinophilic homogenous material. The lamina propria has few lymphocytes and plasma cells. H&E stain, 10X.
(TIF)
