# Correction: Serum 25-hydroxyvitamin D concentrations in dogs with gallbladder mucocele

**DOI:** 10.1371/journal.pone.0258411

**Published:** 2021-10-04

**Authors:** Jared A. Jaffey, Jodi Matheson, Kate Shumway, Christina Pacholec, Tarini Ullal, Lindsay Van den Bossche, Hille Fieten, Randy Ringold, Jung Keun Lee, Amy E. DeClue

The ninth author’s name is incorrect. The correct name is: Jung Keun Lee.
