# Supplementary material for: Serum 25-hydroxyvitamin D concentrations in dogs with gallbladder mucocele
Source: PLoS One. 2020 Dec 16;15(12):e0244102. doi: 10.1371/journal.pone.0244102 (PMC7743984; doi:10.1371/journal.pone.0244102)
Supplement: S1 Table — (DOCX) [file pone.0244102.s001.docx]

**S1 Table.** Breed distribution in dogs with gallbladder mucocele and healthy control dogs.

| Breed | Gallbladder mucocele | Controls |
| --- | --- | --- |
| Australian Cattle Dog | 0 | 1 |
| Beagle | 4 | 0 |
| Bichon Frise | 1 | 0 |
| Border Collie | 1 | 0 |
| Catahoula Leopard | 0 | 1 |
| Cavalier King Charles Spaniel | 2 | 0 |
| Chihuahua | 8 | 0 |
| Chow Chow | 2 | 0 |
| Cocker Spaniel | 2 | 0 |
| Dachshund | 1 | 0 |
| Dogo Argentino | 1 | 0 |
| English Cocker Spaniel | 1 | 0 |
| German Shepherd Dog | 2 | 1 |
| Great Dane | 0 | 1 |
| Jack Russell Terrier | 1 | 0 |
| Labrador Retriever | 2 | 2 |
| Maltese | 3 | 1 |
| Mixed Breed Dog | 13 | 7 |
| Miniature Dachshund | 1 | 1 |
| Miniature Pinscher | 1 | 0 |
| Miniature Poodle | 2 | 0 |
| Miniature Schnauzer | 2 | 0 |
| Parson Russell Terrier | 1 | 0 |
| Pit Bull Terrier | 0 | 2 |
| Pomeranian | 1 | 0 |
| Pug | 1 | 0 |
| Queensland Heeler | 0 | 1 |
| Rat terrier | 1 | 1 |
| Scottish Deerhound | 1 | 0 |
| Shetland Sheepdog | 3 | 0 |
| Shih Tzu | 1 | 0 |
| Springer Spaniel | 2 | 0 |
| Toy Poodle | 2 | 0 |
| West Highland White terrier | 1 | 0 |
| Yorkshire Terrier | 1 | 1 |
