# Supplementary figures and images for: Serum 25-hydroxyvitamin D concentrations in dogs with gallbladder mucocele
Source: PLoS One. 2020 Dec 16;15(12):e0244102. doi: 10.1371/journal.pone.0244102 (PMC7743984; doi:10.1371/journal.pone.0244102)

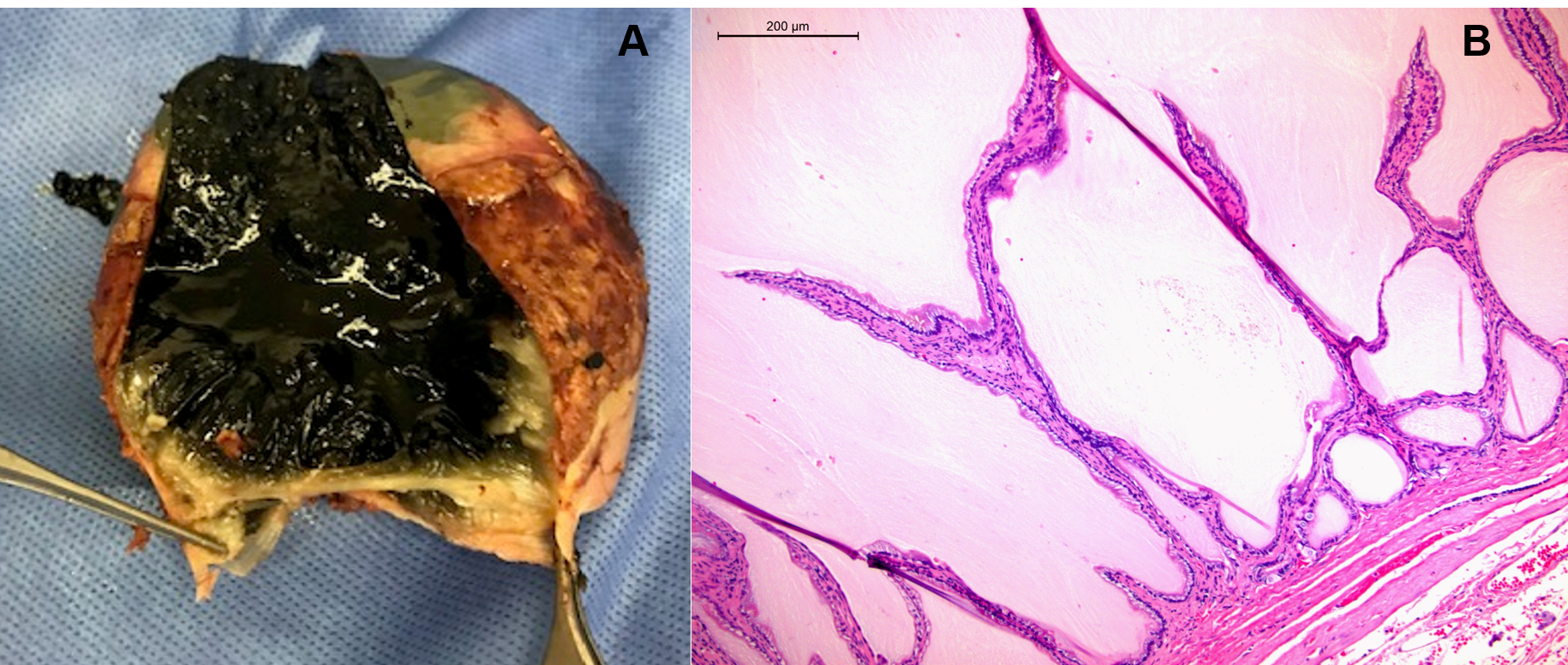

Supplement: S1 Fig — Gross (A) and histopathologic (B) image of a gallbladder mucocele in a dog. (A) The gallbladder is markedly expanded with a large amount of tenacious to gelatinous, mucinous, yellow to dark purple bile; (B) The mucosal epithelial cells are proliferative forming glandular and frond like structures that are lined by cuboidal to tall columnar epithelial cells. The mucosa and the luminal surface are filled with eosinophilic homogenous material. The lamina propria has few lymphocytes and plasma cells. H&E stain, 10X. (TIF) [file pone.0244102.s002.tif]
